# The Clearing of the Slums

**Published:** 1919-06-07

**Authors:** 


### The Clearing of the Slums.

At the annual conference of the National Council of Public Morals and of the National Birth-Rate Commission, Dr. Addison, in opening the discussion on "How to Maintain and Improve the Race," raised a number of points showing of what vast importance in this direction was the great housing campaign which the Government were now starting.

The essential factors were first the clearing and then the reconstruction of the slums. The effort and expense involved would be enormous, but at the same time one heartily agrees with the speaker that without attacking the slum problem at its foundation we cannot expect to produce any progressive or lasting improvement in the physique of the masses concerned. For years a tremendous amount of magnificent and noble-hearted endeavour has been expended by both official and unofficial workers. It is hopeless, however, when there exist thousands upon thousands of so-called homes in which are no proper facilities for ordinary family decency, to expect that we can lastingly improve the physical condition of the people who must, owing to shortage of housing or any other reason, live in them. Building of new houses and the many excellent constructional schemes already in progress all tend to solve one-half of the problem, but for the other half we need organised obliteration of the possibility that people shall ever, either for reasons of necessity or desire, find themselves living and raising families in the absolutely unsanitary dwellings, areas of which are dotted all too profusely over the map of almost any large town in these islands.

